# Pregnancy wantedness, frequency and timing of antenatal care visit among women of childbearing age in Kenya

**DOI:** 10.1186/s12978-016-0168-2

**Published:** 2016-05-04

**Authors:** Rhoune Ochako, Wanjiru Gichuhi

**Affiliations:** Faculty of Medicine and Health Sciences, Ghent University, Ghent, Belgium; Population Studies & Research Institute (PSRI), University of Nairobi, Nairobi, Kenya

**Keywords:** Pregnancy wantedness, Unwanted pregnancy, Mistimed pregnancy, Antenatal care visit, DHS, Kenya, Pregnancy

## Abstract

**Background:**

A woman’s health seeking behaviour during pregnancy has been found to have significant repercussions on her wellbeing and that of her unborn child. For example, the risk of poor pregnancy outcomes and maternal death is higher among women who do not receive antenatal care.

**Methods:**

The study described the characteristics of women who reported wanted, unwanted and mistimed pregnancies from their last birth at the time of the survey; the linkage between frequency of antenatal care visits and pregnancy wantedness and the relationship between timing of the first antenatal care visit and pregnancy wantedness since maternal morbidity and mortality are higher among women who do not receive antenatal care. The 2008-09 Kenya Demographic and Health Survey data is used and multinomial logistic regression and logistic regression informed the study analysis.

**Results:**

Results showed that women, who reported wanted pregnancy were more likely to receive antenatal care while those who reported unwanted pregnancy were less likely to receive antenatal care, but more likely to attend late the first time and have fewer than four antenatal care visits. Also, mistimed pregnancies were associated with low frequency of antenatal care visit and late timing of the first visit.

**Conclusion:**

Our findings confirm an association between pregnancy wantedness, frequency of antenatal care visits and timing of the first antenatal care visit. Women whose pregnancy was reported as mistimed and unwanted were more likely not to receive any antenatal care and when they did; they went for fewer than the recommended four visits with late timing. Health policy and strategies should ensure that all pregnant women regardless of their pregnancy status at the time of conception first receive antenatal care, and receive it in a timely manner and make at least four antenatal care visits before delivery. This will help to identify health complications that may arise during and after delivery and reduce maternal, new-born and infant mortality. Information, education and communication campaigns on family planning especially for spacing and matters related to antenatal care visits, timing and frequency should be intensified nationally.

## Background

A woman’s health seeking behaviour during pregnancy has been found to have significant repercussions on her wellbeing and that of her unborn child [1]. For example, the risk of poor pregnancy outcomes and maternal death is higher among women who do not receive antenatal care (ANC). In many developing countries, women do not attend antenatal care clinics regularly thus missing out on important components of prenatal care such as, health education, screening and diagnosis, treatment and referral. During prenatal care, women at risk of developing complications are identified, where possible interventions are put in place to prevent the development of risks, pre-existing conditions and any complications that arise are also treated. When these are done, the risk of maternal morbidity and mortality are reduced [2]. Monthly antenatal care visits are commended for the first 7 months, thereafter fortnightly during the eighth month and weekly during the last month to delivery [3–5]. WHO (2010) established that essential interventions can be provided over four visits at specified intervals, at least for healthy women with no underlying medical problems. Consequently, WHO defined a new model of ANC based on four goal-oriented visits, that is, what is done in each visits [6]. On the four goal-oriented visits, UNFPA in 2004 explained that the first visit which is expected to screen and treat anaemia, syphilis, screen for risk factors and medical conditions that can be best dealt with in early pregnancy and initiate prophylaxis if required (for example, for anaemia and malaria) is recommended to be held by the end of fourth month. The second, third and fourth visits are scheduled at 24–28, 32 and 36 weeks, respectively [7].

Chuma and Thomas (2013) lament that only a minority of pregnant women (36.1 %) make the required minimum of four ANC visits in public health facilities in Kenya [8]. This implies that majority of women in Kenya are unlikely to get the essential interventions that can be provided over four visits at specified intervals. Kenya National Bureau of Statistics (KNBS) and ICF Macro in 2010 promulgated that Kenya vision 2030 social strategy emphasises the need to improve the overall livelihoods of Kenyans. In the area of maternal health, vision 2030 aims at shifting the health bill from curative to preventive care with special attention being paid to lowering infant and maternal mortality ratios. However, it has been established that women in Kenya do not attend antenatal care clinics regularly, and the country is lagging behind in interventions which should lower infant and maternal mortality [9].

The World Health Organization estimates that about 580,000 women aged 15–49 years die each year from pregnancy related causes with a high proportion of these deaths in sub-Saharan Africa. These deaths are preventable with adequate use of maternal health services [10]. Kenya is not left out in this problem, according to the 2008-09 Kenya Demographic and Health Survey (KDHS) Maternal Mortality Ratio (MMR) was 488 deaths per 100,000 live-births an increase from 444 deaths per 100,000 live births as reported by the 2003 KDHS [9, 11]. Estimates elsewhere put the figure at 1000 deaths per 100,000 live births, representing a 1 in 25 lifetime risk of dying from a maternal-related cause [12]. Although overall use of antenatal care services is reported as high, they are characterised by few visits timed late into pregnancy [13].

Sub-Saharan Africa is grappling with the burden of mistimed and unwanted pregnancy. Pregnancies that were unwanted at the time of conception are associated with a late start of antenatal care and less frequent visits compared to pregnancies that were wanted [14–16]. Similarly, use of antenatal care for mistimed pregnancies is lower on average [14, 15]. Mistimed and unwanted pregnancies have higher chances of resulting into unsafe abortions which may lead to higher maternal morbidity and mortality [14]. A pregnancy is considered unwanted when the woman does not want to have any other child at the time of that pregnancy, while a pregnancy is classified as mistimed if the woman did not want it at the time it occurred but at a later date/time [17, 18]. Mumah et al. (2014) posit that over 40 % of pregnancies in Kenya are unintended; that is either mistimed or unwanted. The high number of unplanned pregnancies stems largely from an unmet need for contraceptives, for instance, Kenya National Bureau of Statistics (KNBS), ICF Macro (2010) postulates that one in four married women in Kenya have an unmet need and the national modern contraceptive prevalence rate is estimated at 39 % [9, 19].

Women whose pregnancies are mistimed or unwanted may be reluctant to initiate antenatal care in the hope that the pregnancy will disappear or in attempts to conceal it. They are therefore less likely to seek antenatal care and when they do, report a late timing of the first antenatal care visit [20–22]. A Study by Dutta *et al* in 2015 show that the risk of experiencing mistimed pregnancy decreases if the woman has higher education. They also found the likelihood of unwanted pregnancy decreases among those women having higher education [23]. Denise et al. in 2004 found that the likelihood of having an unwanted rather than mistimed pregnancy was elevated for women 35 or older (relative risk, 2.3) and was reduced for those younger than 25 (0.8) [24]. Yet another Study by Exavery *et al* in 2014 revealed that young age (<20 years), and single marital status were significant predictors of both mistimed and unwanted pregnancies [25]. They further established that lack of inter-partner communication about family planning increased the risk of mistimed pregnancy significantly. Thus, mistimed or unwanted pregnancy is clearly a public health issue, a gender issue, and a population issue; effectively addressing such a problem will result in multidimensional improvements for Kenyan women and Kenya as a whole.

The current study seeks to describe the characteristics of women who report mistimed or unwanted pregnancies from their last birth at the time of the survey using the 2008-09 Kenya Demographic and Health Survey (KDHS). This study seeks to answer the following objectives;

i. To describe the characteristics of women who report wanted, mistimed and unwanted pregnancies
ii. To determine the linkage between frequency of antenatal care visits and pregnancy wantedness
iii. To establish the relationship between early timing of the first ANC visit and pregnancy wantedness

## Methods

### Source of data

This study used data from the 2008-09 Kenya DHS which is a nationally representative survey of 8444 women aged 15–49. The study used information from the individual women’s questionnaire. Measurement of pregnancy wantedness is based on questions about the desirability of recent pregnancies reported. The question asked to women was as follows *“At the time you became pregnant with (NAME), did you want to become pregnant then, did you want to wait until later, or did you not want to have another (more) children at all?”*, the response was classified into three categories, wanted then, wanted no more and wanted later. This study was limited to the responses of 4014 women given the most recent birth of each woman aged 15–49 years at the time of the last birth. Information on the most recent births was used as it was assumed that the woman could vividly remember events during pregnancy.

### Study variables

The dependent variables included timing of the first ANC visit, and frequency of ANC visits. The first dependent variable, timing of first ANC visit was grouped as *“None”* for women who did not receive ANC; *“Late”* for women who received the first ANC visit either in the second or third trimester; or *“Early”* for women who received the first ANC visit in the first trimester. The second dependent variable, frequency of ANC visits was a count variable, grouped as a bivariate variable; 4 or more visits, or less than 4 visits/no ANC. The primary independent variable was pregnancy wantedness and socio-economic and demographic variables of interest in the study were education, household wealth, place of residence, ethnicity, parity, preceding birth interval, maternal age, working status and marital status.

### Methods of analysis

The methods of analysis used were multinomial logistic regression and linear regression. Data analysis was carried out using STATA v.14, where descriptive statistics were used to provide sample characteristics. The first outcome variable, timing of the first ANC visit was analysed using bivariate and multinomial logistic regression since it was a three outcome variable coded as none, late and early. We fit one model to predict the linkage between pregnancy wantedness and timing for the first ANC visit in the presence of other explanatory variables. The second outcome variable, frequency of antenatal care visits is analysed using bivariate and multivariate logistic regression to determine the relationship with pregnancy wantedness in the presence of other explanatory variables.

## Results

### Characteristics of study population

Table 1 showed the descriptive analyses of a sample of 4014 women aged 15–49. Slightly more than half of the women (58 %) had wanted pregnancies while 24 % and 18 % had mistimed and unwanted pregnancies, respectively. About 73 % were rural residents while 56 % had primary education with the rest, 18 % and 25 % reporting no education and at least secondary education, respectively. Only 14 % of the women attended their first ANC visit in the first trimester and only 46 % made the recommended four ANC visits.

**Table 1:** Percentage distribution of variables in the sample included in the analysis, women, 15–49, Kenya 2008/09 [Unweighted]

Characteristics	%	Number of cases
Timing of first ANC		
None	11.1	445
Late	75.0	3011
Early	13.9	558
Frequency of ANC		
None	11.1	445
1–3 visits	43.3	1740
4+ visits	45.6	1829
Pregnancy wantedness		
Wanted	58.3	2341
Mistimed	24.0	964
Unwanted	17.7	709
Region		
Nairobi	8.2	328
Central	9.8	393
Coast	14.3	574
Eastern	12.9	517
Nyanza	17.6	708
Rift Valley	17.0	683
Western	12.6	504
North Eastern	7.7	307
Residence		
Rural	73.3	2941
Urban	26.7	1073
Education		
None	18.4	739
Primary	56.2	2257
Secondary or higher	25.4	1018
Household wealth		
Poor	40.9	1641
Medium	32.6	1309
Rich	26.5	1064
Marital status		
Never married	8.6	347
Currently married	82.3	3305
Formerly married	9.1	362
Age		
15–24	34.8	1397
25–34	45.1	1811
35–49	20.1	806
Parity		
1	21.8	878
2–4	50.5	2026
5 and above	27.7	1110
Working status		
No	43.9	1762
Yes	56.1	2252
Preceding birth interval		
First borns	21.8	878
Less than 25 months	18.0	721
25–36 months	24.2	972
37+ months	36.0	1443
Total (N)	100.0	4014

With regard to region of residence, 8 % of the women were each from Nairobi and North Eastern 10 % from Central, 13 % each were from Eastern and Western, while 17 % and 18 % were from Rift Valley and Nyanza, respectively. Majority of the women (45 %) were aged 25–34 years and the rest 35 % and 20 % were aged 15–24 and 35–49 years respectively. Majority of the women were currently married (82 %), half (51 %) had 2 and 4 children and 18 % had a preceding birth interval of less than 25 months.

### Association with timing of the first ANC visit

Bivariate results presented in Table 2 (Col. 1 and 2) show that women with unwanted pregnancies were more likely not to receive ANC (*p* < 0.001 in Col.1) and when they did, the visits were late (*p* < 0.05 in Col.2). Considering region of residence, Women from Rift Valley were (2.6 times, *p* < 0.05 in Col.1) and North Eastern (13.4 times, *p* < 0.001 in Col.1) more likely not to receive ANC compared to their counterparts from Nairobi. On the other hand, women from Central, Coast and Eastern were more likely to receive ANC late (*p* < 0.05 in Col.2) compared to women from Nairobi. Compared to rural women, urban women were less likely not to receive ANC (*p* < 0.001 in Col.1), similarly, they were less likely (*p* < 0.01 in Col. 2) to receive ANC late. As expected, women with no education were 3.7 times (*p* < 0.001) more likely not to receive ANC compared to those with primary education. Women with at least secondary education were less likely not to receive ANC (*p* < 0.001 in Col.1), likewise, they were less likely to receive it late (*p* < 0.01 in Col.1 and 2). Older women, 35–49 years, were more likely (*p* < 0.01) not to receive ANC compared to their younger counterparts aged less than 25 years.

**Table 2:** Odds ratio based on multinomial logistic regression analysis, of timing of the first antenatal care visit among women, 15–49, Kenya 2008/09

	Panel A: Bivariate		Panel B: Multivariate	
	None vs Earl*y*	Late vs Early	None vs Early	Late vs Early
	Col. 1	Col. 2	Col. 3	Col. 4
Pregnancy wantedness				
Wanted	1.00	1.00	1.00	1.00
Mistimed	1.06	1.30	1.02	1.15
Unwanted	2.45***	1.46*	1.75*	1.11
Region				
Central	2.22	2.04*	0.98	1.57
Coast	1.98	1.78*	0.36	1.26
Eastern	2.10	1.79*	0.46	1.01
Nyanza	2.11	0.72	0.57	0.97
Rift Valley	2.60*	0.38	0.45	0.80
Western	2.42	1.70	0.58	0.88
North Eastern	13.41***	2.02	1.30	1.24
Residence				
Rural	1.00	1.00	1.00	1.00
Urban	0.26***	0.61**	0.54	1.10
Education				
Primary	1.00	1.00	1.00	1.00
None	3.69***	0.82	3.02 **	0.63
Secondary or higher	0.31***	0.57	0.47 **	0.76
Household wealth				
Poor	1.00	1.00	1.00	1.00
Medium	0.37***	0.80	0.57*	0.77
Rich	0.16***	0.41***	0.42*	0.43**
Marital status				
Currently married	1.00	1.00	1.00	1.00
Never married	2.25*	1.19	4.33***	1.43
Formerly married	1.41	1.09	1.26	1.05
Age				
15–24	1.00	1.00	1.00	1.00
25–34	0.77	0.97	0.60*	0.94
35–49	2.03**	1.15	0.75	0.83
Parity				
1	1.00	1.00	1.00	1.00
2–3	1.10	1.11	1.66	1.13
4 and above	3.81***	1.88***	3.81**	1.75*
Working status				
No	1.00	1.00	1.00	1.00
Yes	1.01	0.95	1.19	0.93
Preceding birth interval				
37+ months	1.00	1.00	1.00	1.00
First borns	0.79	0.69	0.71	0.85
Less than 25 months	0.66	0.40***	1.27	1.30
25–36 months	0.79	0.67	1.00	1.00

*p < .05; **p < .01 ***p < .001

Considering wealth status of a household, those from medium and high wealth households were less likely (*p* < 0.001 in Col.1) not to receive ANC than those from poor households. Women with 4 or more children were more likely not to receive ANC than those who had one child.

### Determinants of timing of the first antenatal care visit

Table 2, Panel B (Col.3 and 4) provide odds ratio based on multinomial logistic regression analysis, for timing of the first antenatal care visit among women, 15–49 years. This model confirms what was observed in the bivariate model where women with unwanted pregnancy remained more likely not to receive ANC (*p* < 0.05 in Col.3) compared to those who reported their pregnancy as wanted/timed. As expected, women with no education were more likely not to receive ANC (*p* < 0.01 in Col.3) while those with at least secondary education were less likely not to receive ANC (*p* < 0.01 in Col.3) compared to those with primary education. Women from middle and rich households were less likely not to receive ANC (*p* < 0.05). On the other hand, women from rich households were less likely not to receive ANC late (*p* < 0.01 in Col.4) compared to those from poor households. Never married women (*p* < 0.001) were 4.3 times more likely not to receive ANC while those aged 25–34 years (*p* < 0.05) were less likely not to receive ANC compared to those under 25 years. Women with 4 children and above were more likely not to receive ANC and when they did it was timed late.

### Association with frequency of antenatal care visits

Table 3 presents bivariate results of logistic regression of frequency of ANC among women aged 15–49 years (Col.1). The results show that women with mistimed and unwanted pregnancies were 37 % and 41 % respectively less likely to go for at least 4 ANC visits (*p* < 0.001) compared to those whose pregnancy was reported as wanted. Considering region of residence, women from Coast, Eastern, Nyanza, Rift Valley, Western and North Eastern were all less likely to receive at least 4 ANC visits compared to their counterparts from Nairobi. Urban women were 1.9 times (*p* < 0.001) more likely to go for at least 4 ANC visits compared to their rural counterparts. Considering level of education, women with no education were less likely to go for at least 4 ANC visits while those with at least secondary education were more likely to go for 4 or more ANC visits. Women aged 25–34 years were more likely to go for 4 or more ANC visits than those less than 25 years. Additionally, those with four or more children were less likely to go for 4 or more ANC visits than those with one child. Considering the preceding birth interval, women whose interval was less than 25 months or those whose birth was the first (first born) were less likely to go for 4 or more ANC visits compared to those whose interval was 37 months and more.

**Table 3:** Odds ratio of logistic regression analysis, of frequency of antenatal care visits among women, 15–49, Kenya 2008/09

	Bivariate - Col.1	Multivariate - Col. 2
Pregnancy wantedness		
Wanted	1.00	1.00
Mistimed	0.63 ***	0.73**
Unwanted	0.59***	0.69**
Region		
Nairobi	1.00	1.00
Central	0.72	0.90
Coast	0.49**	0.83
Eastern	0.48***	0.81
Nyanza	0.39***	0.65*
Rift Valley	0.41***	0.70
Western	0.36***	0.65*
North Eastern	0.25***	0.59
Residence		
Rural	1.00	1.00
Urban	1.88***	0.86
Education		
Primary	1.00	1.00
None	0.73*	0.83
Secondary or higher	2.36***	1.86***
Household wealth		
Poor	1.00	1.00
Medium	1.44**	1.22
Rich	2.84***	1.92***
Marital status		
Currently married	1.00	1.00
Never married	0.76	0.73
Formerly married	0.93	0.96
Age		
15–24	1.00	1.00
25–34	1.36**	1.23
35–49	1.08	1.21
Parity		
1	1.00	1.00
2–3	0.98	1.03
4 and above	0.64***	0.88
Working status		
None	1.00	1.00
Yes	1.17	1.08
Preceding birth interval		
37+ months	1.00	1.00
First borns	0.66**	0.81
Less than 25 months	0.72*	0.84
25–36 months	1.05	1.00

*p < .05; **p < .01 ***p < .001

### Determinants of frequency of antenatal care visits

Table 3 Col. 2 provides the results of multivariate logistic regression of frequency of ANC visits among women aged 15–49 years. This model confirms that women with mistimed (27 %) and unwanted pregnancy (31 %) were less likely (*p* < 0.01 in Col.2) to receive 4 or more ANC visits than those whose pregnancies were wanted. Women from Nyanza and Western were 35 % less likely to receive 4 or more ANC visits than their Nairobi counterparts. Considering household wealth, those from high wealth households were more likely to go for 4 or more ANC visits than those from poor households. As expected, women with secondary or higher education were more likely to go to 4 or more ANC visits compared to those with primary education.

## Discussion

This study sought to describe the characteristics of women who reported wanted, mistimed or unwanted pregnancies, to determine the linkage between frequency of ANC and pregnancy wantedness, and to establish the relationship between early timing of the first ANC visit and pregnancy wantedness. Results in both bivariate and multivariate multinomial analysis show that women who reported wanted pregnancy were more likely to receive ANC while those who reported unwanted pregnancy were less likely to receive ANC. These finding is similar to those of Dutta *et al* in 2015, Exavery *et al* in 2014 and Denise et al. in 2004 who also report early timing for women reporting their pregnancy as wanted [23–25] On the other hand, women who reported mistimed pregnancies were more likely to receive late ANC while those who report wanted pregnancies were more likely to receive early ANC.

The study established that certain aspects of region of residence, place of residence, marital status, level of education, household wealth, age of the woman, parity, and preceding birth interval were all associated with frequency and timing of ANC visits. Controlling for other factors, region had a significant association with timing and frequency of ANC visits. Women from North Eastern were particularly more likely not to receive ANC compared to those from Nairobi. On the other hand, women from Coast, Eastern, Western Nyanza, Rift Valley and North Eastern were all less likely to receive 4 or more ANC visits compared to their counterparts from Nairobi.

As expected, place of residence was found to be associated with timing of the first ANC visit and receiving the service. Women living in urban areas were more likely to receive ANC and to receive early ANC service compared to their rural counterparts. Furthermore, education was found to be associated with timing of the first ANC. Women with secondary and above education were more likely to go for 4 or more ANC visits. Also, women from rich households were more likely to go for 4 or more ANC visits and to receive it early compared to those from poor households. These findings are similar to those of a study in rural Kenya that reported the advantages of more than 8 years of education to utilization of antenatal care [26]. Results on marital status confirmed that never married women were less likely to receive ANC as compared to those who were currently married and if they did receive, it was timed late compared to those who were currently married. It has also been established that age was associated with receiving ANC and timing of the first visit. Older women (35–49 years) were more likely to receive no ANC as compared to younger women (15–24 years). Most of the time, younger women who are unmarried hid their pregnancy to avoid potential social implications of the pregnancy and thereby delaying initiation of antenatal care [27]. The results also established that parity was associated with receiving ANC and timing of the first ANC. Women with more than four children were more likely not to receive ANC and if they received any they received it late compared to those of parity one. Finally the multivariate results showed that women whose preceding birth was less than 37 months were more likely not to receive ANC and more likely to receive late care as compared to those whose preceding birth was 37 months and more.

On the other hand, the results in the logistic regression of the frequency of ANC showed that women with unwanted pregnancies were less likely to go for 4 or more ANC as compared to those with wanted pregnancies. Results showed that region of residence was related to the frequency of attending ANC. Women from North Eastern were less likely to attend more ANC visits compared to women from Nairobi. Both bivariate and multivariate results showed that women living in urban were more likely to attend more ANC visits compared to their rural counterparts. Women with secondary education were more likely to attend more ANC visits compared to those with primary education while those with no education were less likely to attend more ANC visits compared to those with primary education. Wealth status was associated with frequency of ANC visits, where women from medium and rich households were more likely to go for more ANC visits compared to those from poor households. Considering marital status, never married women were disadvantaged in terms of receiving more ANC visits compared to those who were currently married.

In regard to age, women aged 25–34 years were more likely go for more ANC visits compared to those aged 15–24 years. A study conducted in Ghana and Kenya also found younger women to be less likely to visit ANC early, most of the time, these younger women were uncertain about their pregnancy status thereby causing delays in utilisation of care [27].

Higher parity women were less likely to go for more ANC visits compared to parity one women, similar results were observed among women whose birth interval was less than 37 months who were less likely to go for more ANC visits compared to those whose preceding birth interval was at least 37 months. Finally, women in employment were more likely to go for more ANC visits compared to their counterparts who were never working.

## Conclusion

The results from this study are a clear pointer on the influence of pregnancy wantedness on utilization of antenatal care services. Targeted interventions to emphasize the importance of early timing and having at least 4 ANC visits remains important to both women who report wanted and unintended (mistimed and unwanted) pregnancies. The disparities in utilization of antenatal care services at regional and residence point to the needs and vulnerability of women from these areas. The Ministry of Health should have targeted interventions in these areas to ensure disadvantaged women benefit. Additionally, this study highlights predictors of antenatal care use, thereby identifying vulnerable groups. This information is useful in developing targeted interventions that will increase utilization of reproductive health services. Targeted health policies that promote the use of antenatal care services among all pregnant women regardless of their pregnancy status at the time of conception where they receive ANC, and receive it in a timely manner and attend at least four ANC visits before delivery. This will help to identify health complications that may arise during and after delivery and to reduce maternal, new-born and infant mortality. Information, education and communication campaigns on family planning especially for spacing and matters related to ANC visits, timing and frequency should be intensified nationally.
